# IL-6/JAK2/STAT3 axis mediates non-classical monocyte-induced Th17-like conversion of activated Tregs in acute myeloid leukemia patients

**DOI:** 10.1186/s12885-026-16619-6

**Published:** 2026-07-28

**Authors:** Xiaoran Dai, Yuanling Zheng, Xu Liu, Ruoyu Huang, Mei Fu, Meijuan Zheng, Yuanhong Xu, Jianyao Huang, Lu Zong

**Affiliations:** 1 Department of Clinical Laboratory, The First Affiliated Hospital of Anhui Medical University, #218 Jixi Road, Hefei, Anhui 230032 China; 2 Clinical Medical College of Anhui Medical University, Hefei, Anhui China; 3 Department of Blood Transfusion, The Third People’s Hospital of Datong, Datong, Shanxi China; 4 Anhui Medical University, Hefei, Anhui China; 5 Department of Hematology, The First Affiliated Hospital of Anhui Medical University, #218 Jixi Road, Hefei, Anhui 230032 China

**Keywords:** Activated Tregs, Th17-like phenotype, IL-6/JAK2/STAT3 pathway, AML, Tumor escape

## Abstract

**Purpose:**

Regulatory T cells (Tregs) promote tumor escape. Here we show CD45RA^−^FoxP3^high^CD4^+^ activated Tregs (aTreg, Fr. II) acquired a Th17-like phenotype in acute myeloid leukemia (AML). The primary objective of this study was to explore the specific roles of non-classical monocytes in regulating the increased Fr. II plasticity to Th17-like phenotype to form the immunosuppressive microenvironment.

**Methods:**

We stratified phenotypically heterogeneous FoxP3⁺CD4⁺ T cells from AML patients into three subsets based on FoxP3 and CD45RA, aiming to dissect their functional diversity by flow cytometry. Flow cytometric sorting, co-culture assays, in vitro neutralization experiments, and JAK2 blockade assays were employed to demonstrate that non-classical monocytes induce Th17-like Fr. II cells through the IL-6/JAK2/STAT3 pathway.

**Results:**

CD45RA^−^FoxP3^high^ Fr. II, which performed a major inhibitory role in three FoxP3⁺CD4⁺ T subsets, were reduced in numbers but enhanced their immunosuppressive capacity in AML. Besides, Fr. II had a Th17-like phenotypic shift (marked by augmented IL-17 A and RORγt expression). Co-culture assays and in vitro neutralization experiments demonstrated that IL-6 secreted by non-classical monocytes regulates the conversion of Fr. II toward a Th17-like phenotype. Furthermore, JAK2 inhibitor reduced the phosphorylation of STAT3 and impaired Th17-like differentiation potency of Fr. II in AML patients. Notably, IL-17 A⁺ Fr. II were reduced after treatment.

**Conclusions:**

Taken together, we found that non-classical monocytes drive the differentiation of Th17-like Fr. II via the IL-6/JAK2/STAT3 signaling pathway in AML. IL-17 A⁺ Fr. II may correlate with disease activity. Our finding reveals a new immune-evasion mechanism in AML, and offers a potential therapeutic target in the immunotherapy of AML.

**Graphical Abstract:**

**Figure:**
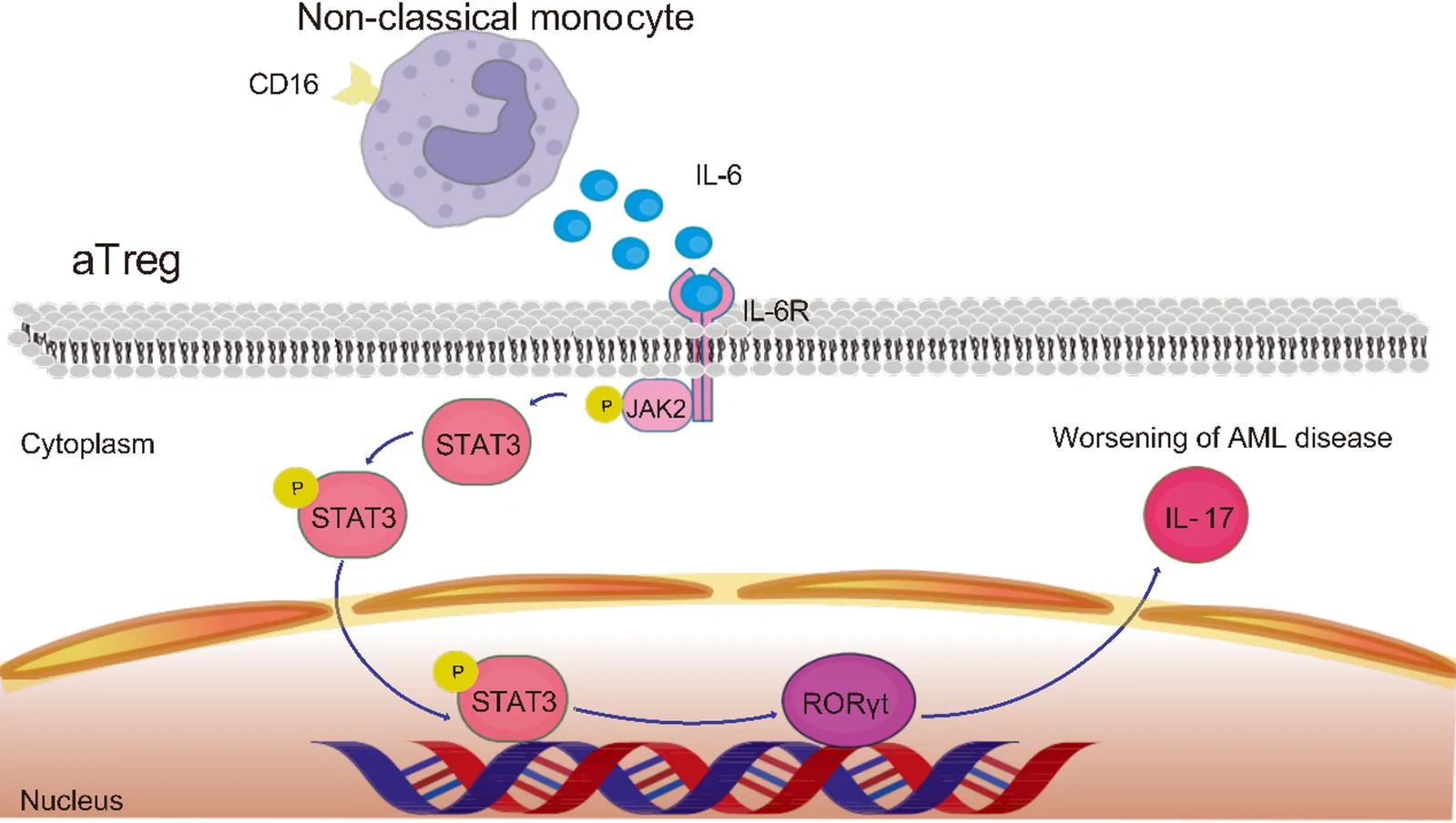
IL-6/JAK2/STAT3 axis mediates non-classical monocyte-induced Th17-like conversion of activated Tregs in acute myeloid leukemia patients. This study demonstrates that CD14^-^CD16^+^ nonclassical monocytes secrete IL-6, which binds to IL-6R on aTregs. This triggers downstream JAK2 phosphorylation, followed by STAT3 phosphorylation and subsequent RORγt expression, thereby stimulating aTregs to secrete IL-17 and further aggravating the severity of AML.

**Supplementary Information:**

The online version contains supplementary material available at https://doi.org/10.1186/s12885-026-16619-6.

## Introduction

Acute myeloid leukemia, a clonal malignant hematological disease, establishes an immunosuppressive environment in which both innate and adaptive immune responses are disrupted [1, 2]. Emerging evidence suggests that various immune cells promote this imbalanced immune state to sustain leukemic cell survival. The immune microenvironment in both bone marrow and peripheral blood of AML shows the accumulation of immunosuppressive regulatory T cells (Tregs) [3, 4]. In particular, an elevated frequency of Tregs is linked to poor prognosis in AML patients [5]. A relatively high percentage of CD4^+^CD25^high^Foxp3^+^ Tregs upregulated the expression of CTLA-4 and GITR [6]. Tregs can regulate the expansion of leukemia stem cells (LSCs) and leukemia progenitor cells through intercellular interactions or cytokine secretion, and assist leukemia cells in immune escape [3]. Myeloid-derived suppressor cells (MDSCs) are also more abundant in AML patients and contribute to tumor-associated immune suppression [7]. Notably, scRNA-seq analysis revealed an imbalance in the differentiation of CD4^+^ T cells in AML patients, and these cells tend to differentiate into Th17-like cells, which may also be a key mechanism of immune escape in AML [8]. In addition to traditional chemotherapy, emerging immunotherapies have been designed to neutralize the effects of the immunosuppressive environment [2]. However, immunosuppressive cells form an immune-privileged niche that serves as a refuge for leukemia cells, hampering the therapeutic efficacy of immunotherapies. Therefore, investigations into the pathogenesis of AML are urgently needed to develop new immunotherapy interventions.

Human FoxP3^+^CD4^+^ T cells contain three subgroups with different phenotypes and functions: CD45RA^+^FoxP3^low^ resting Treg cells (rTregs, Fr. I), CD45RA^−^FoxP3^high^ activated Treg cells (aTregs, Fr. II), and CD45RA^−^FoxP3^low^ nonsuppressive T cells (Fr. III), which have no inhibitory function [9–11]. rTreg cells can proliferate and transform into aTreg cells rapidly as terminally differentiated aTreg cells die [9, 12]. Importantly, of the three FoxP3^+^CD4^+^ T-cell subsets, Fr. II cells performed a major inhibitory role [13]. In contrast, increasing evidence suggests that Fr. III cells are a heterogeneous group of memory/effector T cells that respond rapidly to promote antitumor responses [10, 14]. Collectively, these results highlight that human FoxP3^+^CD4^+^ cells contain inhibitory/noninhibitory populations. The investigation of the functional heterogeneity and differentiation dynamics of FoxP3^+^CD4^+^ T cells allows us to better define immunomodulatory functions in different diseases.

Th17 cells promote immunity against infection through the secretion of cytokines and Th1 cell recruitment. The balance of Tregs and Th17 cells influences the outcome of the host immune response to pathogen infection. Notably, Tregs acquire phenotypic and functional plasticity toward Th17 cells [15, 16]. Tregs derived from the peripheral blood of patients with severe psoriasis tend to differentiate into Th17-related phenotypes driven by IL-23 [17]. During *Mycoplasma pneumoniae* infection, Fr. II gradually reduced its inhibitory function and evolved into a Th17-like phenotype. The level of IL-17 A secreted by Fr. II increases significantly, which ultimately promotes the development of drug rashes or asthma [18]. However, the signaling pathway by which Tregs express Th17-related cytokines and its clinical significance remain unknown.

Previous studies have shown that the proportion of Tregs in the peripheral circulation of AML patients is significantly greater than that in the peripheral circulation of healthy people and that Th17 cells are also increased [6, 19]. Moreover, the elevated levels of Th17 and Treg cells are positively correlated with AML onset [20]. This study revealed that although CD25^+^FoxP3^+^CD4^+^ T cells were increased in AML patients, the proportion and number of Fr. II exerting a central suppressive role decreased. Fr. II enhance their immunosuppressive function and develop into a Th17-like phenotype in the presence of IL-6 expressed by non-classical monocytes through the JAK2/STAT3 signaling pathway. Importantly, AML patients with a greater proportion of IL-17 A^+^ Fr. II had a poor prognosis. Overall, this study clarified a new pathogenesis of AML, which provides a new therapeutic target for AML patients.

## Materials and methods

### Patients and clinical samples

Inclusion criteria were AML patients with a clinically confirmed diagnosis who were in the prechemotherapy stage were collected from the First Affiliated Hospital of Anhui Medical University, from July 2022 to July 2026. Exclusion criteria included patients with autoimmune diseases or other malignancies. Healthy control (HC) samples were sourced from the hospital’s physical examination center and tested normally for blood laboratory indices. The clinical parameters of the HCs and AML patients included in this study are summarized in Supplementary Table 1. Both male and female subjects were included, with ages ranging from 18 to 86 years. Written informed consent was provided by all participants.

Peripheral blood mononuclear cells (PBMCs) were isolated from peripheral blood via Ficoll (TBD Science, China) density gradient centrifugation for lymphocyte detection or via red blood cell lysis buffer (Solarbio, China) treatment for monocyte detection. Bone marrow mononuclear cells (BMMCs) were isolated from bone marrow via red blood cell lysis buffer. The isolated PBMCs and BMMCs were labeled with antibodies for flow cytometry analysis.

### Laboratory index detection

Peripheral blood was collected in EDTA anticoagulant tubes and analyzed via XE2100 (Sysmex, Japan) with corresponding reagents for testing indicators such as white blood cells, platelets, hemoglobin, lymphocytes, monocytes, and neutrophils.

### Flow cytometry analysis

For surface marker detection, isolated PBMCs and BMMCs were stained with antibodies, washed and detected via a flow cytometer (BD FACSCanto plus). For detection of intracellular cytokine production, PBMCs and BMMCs were stimulated with PMA (50 ng/mL; Multisciences, China) and ionomycin (1 µg/mL; Sigma‒Aldrich, USA) in 5% CO_2_ at 37 °C for 4 h. Monensin (2.5 µg/mL; Multisciences, China) was added during the last hour of stimulation. Then, the cells were washed, labeled with surface molecular antibodies, fixed, permeabilized, stained with intracellular molecular antibodies, and finally detected by a flow cytometer. The data were analyzed via FlowJo 10.8.1. The antibodies used were as follows: BV605-anti-CD4, PE-CY7-anti-CD4, FITC-anti-CD45RA, PE-anti-CD25, PerCP-Cy5.5-anti-HLA-DR, APC-anti-CD20, PE-CY7-anti-CD14, 7-AAD, FITC-anti-Annexin V, BV421-anti-Ki-67, BV421-anti-IL-6, BV421-anti-PD-1, BV450-anti-Bcl-2, BV421-anti-IL-21, BV421-anti-IL-2, PerCP-Cy5.5-anti-IFN-γ and PE-anti-RORγt, which were obtained from BD Biosciences. The APC-CY7-anti-CD45RA, PerCP-Cy5.5-anti-CD25, APC-anti-CD3, Alexa Fluor^®^ 647-anti-CD56, APC-anti-CD19, APC-anti-CD66b, BV510-anti-CD16, PE-CY7-anti-TGF-β1, APC-CY7-anti-IL-17 A, AF488-anti-IL-17 A, and BV605-anti-TIGIT antibodies were obtained from BioLegend. The eFluor^®^ 660 anti-FoxP3 antibody was from eBioscience.

### Phospho-flow analysis

First, the PBMCs were stained with anti-CD4 and anti-CD45RA antibodies at 4 °C for 30 min, fixed with prewarmed fixation buffer (BioLegend, USA) at 37 °C for 15 min, and permeabilized with prechilled True-Phos™ Perm Buffer (BioLegend, USA) at -20 °C for 60 min. Then, the cells were washed and stained with anti-FoxP3 at 4 °C for 1 h and PE-conjugated anti-phospho-Stat3 (pY705) (BioLegend, USA) at room temperature for 30 min. Finally, the cells were washed, resuspended and analyzed with a flow cytometer.

### Assays to measure Fr. II suppression

Fr. II (FoxP3^high^CD45RA^−^CD4^+^) cells were prepared by sorting CD25^+++^CD45RA^−^CD4^+^ T cells via a BD FACSAria II flow cytometer as previously described [9, 12]. Moreover, responder T cells (Tresp), which were sorted as CD25^−^CD45RA^+^CD4^+^ T cells, were labeled with 1 µM carboxyfluorescein diacetate succinimidyl ester (CFSE, Biolegend, USA) and cocultured with Fr. II at 37 °C and 5% CO_2_ at a ratio of 5:1 for 5 days. The coculture system in 96-well round-bottom plates included 90% RPMI 1640 medium (Gibco, USA), 1.5% HEPES (Gibco, USA), 1% streptomycin/penicillin (Beyotime, China), 10% fetal bovine serum (FBS, Gibco, USA), and 500 IU/mL recombinant human IL-2 (PeproTech, USA), together with anti-human CD3 (5 µg/mL; Biolegend, USA), which was plated overnight prior to treatment and discarded the next day, and anti-human CD28 (1 µg/mL; BD Biosciences, USA). After incubation, the cells were washed, harvested, stained with surface markers and assessed for the CFSE signal of Tresp by flow cytometry. The suppressive capacity of Fr. II toward Tresp was calculated via the following formula as described previously [21]:

$$\begin{array}{c}\mathrm{Suppression}\;\left(\%\right)=\left[100\times\left(1-\frac{\%\mathrm{CFSE}^{\mathrm{low}}\mathrm{CD}4^+\mathrm{CD}25^-\;\mathrm T\;\mathrm{cells}\;\mathrm{in}\;\mathrm{co}-\mathrm{culture}}{\%\mathrm{CFSE}^{\mathrm{low}}\mathrm{CD}4^+\mathrm{CD}25^-\;\mathrm T\;\mathrm{cells}\;\mathrm{alone}}\right)\right]\end{array}$$

### *In vitro* neutralization assay and JAK2 blocking assay

First, isolated PBMCs or sorted cells were stimulated with or without anti-CD3/28 for 72 h. Neutralizing antibodies against IL-6 (10 µg/mL; BioXcell, USA), TGF-β (10 µg/mL; BioXcell, USA), or a JAK2 inhibitor (100 nM; Selleckchem, USA) were added to the corresponding wells according to the experimental design, along with appropriate isotype antibodies (mouse/rat IgG) or DMSO. Finally, the cells were washed, harvested and stained with antibodies for flow cytometry.

### Coculture assay of CD3^+^ T cells and monocytes

To verify the role of monocytes, Lin (CD3, CD56, CD66b, CD19, CD20)^−^HLA-DR^+^CD14^−^CD16^+^ non-classical monocytes sorted from AML patients and CD3^+^ T cells sorted from healthy individuals were cocultured at a ratio of 1:10 with anti-CD3/28 stimulation in 96-well round-bottom plates for 72 h.

Furthermore, to verify the effect of IL-6 secreted by non-classical monocytes in AML patients, we sorted non-classical monocytes from AML patients and CD3^+^ T cells from HCs and cocultured them as mentioned above. Next, the cocultured cells were treated with anti-IL-6 neutralizing antibodies or a JAK2 inhibitor for 72 h. An isotype antibody or DMSO was added to the corresponding wells. Finally, the cocultured cells were harvested, washed and stained with antibodies for flow cytometry.

### Statistical analysis

All the data were analyzed via GraphPad Prism 9.4.1 software and are presented as the means ± SEM. For comparisons between two groups, based on the normal distribution test (Shapiro–Wilk test), either Student’s *t* test or the Mann–Whitney *U* test was applied. A statistically significant difference was defined as *P* < 0.05.

## Results

### CD45RA⁻FoxP3^high^ (Fr. II) cells are significantly reduced in the peripheral blood of AML patients

**Fig. 1:**
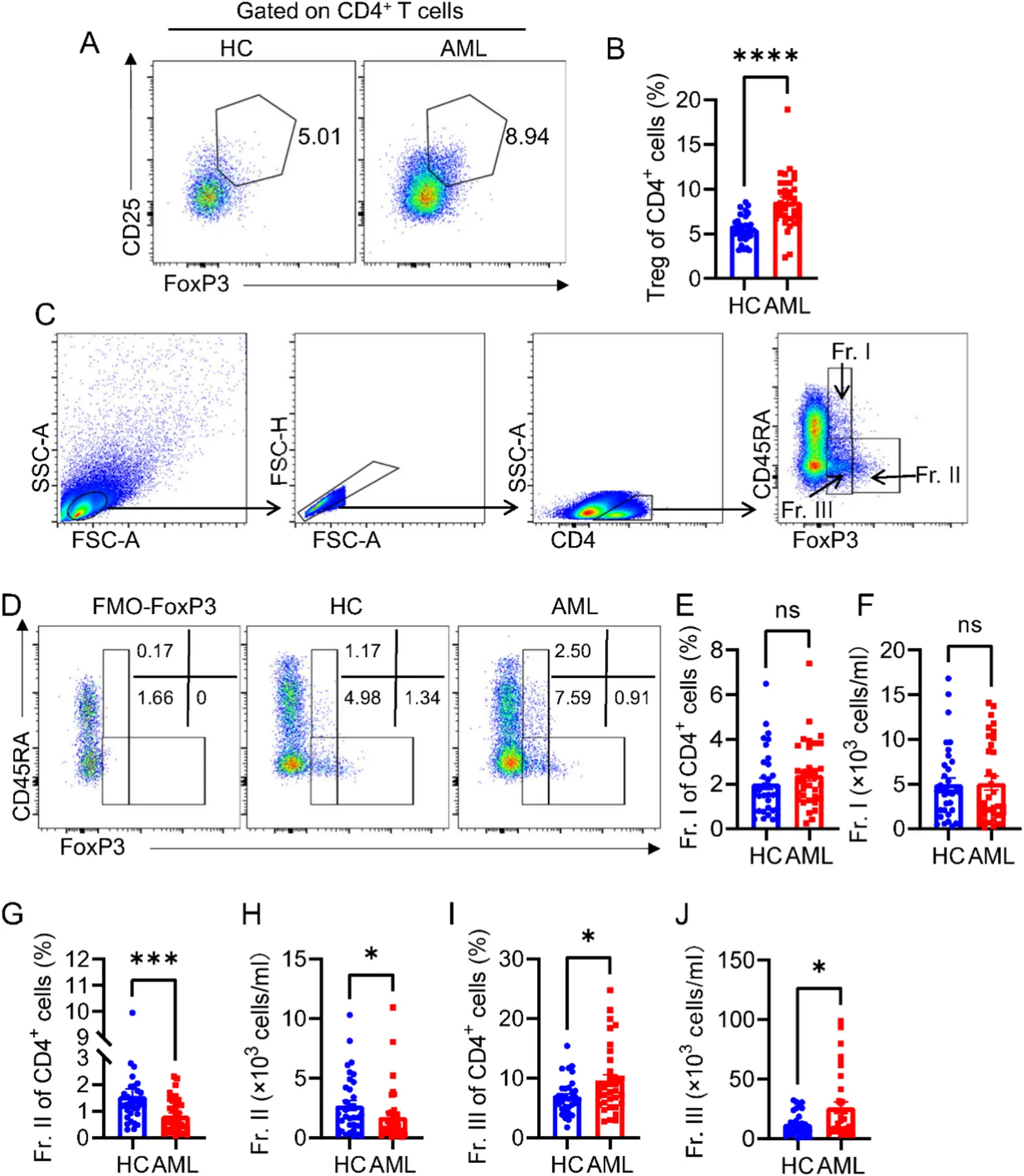
CD45RA^−^FoxP3^high^ (Fr. II) cells are significantly reduced in AML patients. **A**, **B **Representative flow cytometry plots and the average percentages of Foxp3^+^CD25^+^ Tregs among CD4^+^ T cells in healthy controls (HCs) and acute myeloid leukemia (AML) patients (*n* = 34 in each group). **C **Gating strategies for three subsets of Foxp3^+^CD4^+^ T cells (Fr. I: CD45RA^+^Foxp3^low^ Tregs, Fr. II: CD45RA^−^FoxP3^high^ Tregs, and Fr. III: CD45RA^−^Foxp3^low^CD4^+^ T cells) are illustrated. **D **Flow cytometry plots, including one FMO-Foxp3 control plot and two representative plots depicting three Foxp3^+^CD4^+^ T cell subsets in HCs and AML patients respectively, are shown. **E**-**J **The proportions and absolute cell count of (**E**, **F**) Fr. I, (**G**, **H**) Fr. II and (**I**, **J**) Fr. III in HCs and AML patients (*n* = 34 in each group). The data are expressed as the means ± SEM. The Mann–Whitney *U* test was employed for statistical analysis. *P < 0.05; ***P < 0.001; ****P < 0.0001; ns, not significant

Patients with clinically confirmed AML and healthy controls (HCs) were enrolled in this study. First, the proportion of CD25⁺FoxP3⁺CD4⁺ Tregs in the peripheral blood of AML patients was greater than that in the peripheral blood of HCs (Fig. 1A-B), which is consistent with the findings of previous studies [5, 6]. Research has shown that FoxP3⁺CD4⁺ T cells consist of three phenotypically and functionally distinct subsets: CD45RA⁺FoxP3^lo^ resting Treg cells (Fr. I), CD45RA⁻FoxP3^hi^ activated Treg cells (Fr. II), and cytokine-secreting CD45RA⁻FoxP3^lo^ nonsuppressive T cells (Fr. III) (Fig. 1C) [9–11]. The proportion and absolute count of Fr. II, which exert the major suppressive function, were decreased significantly in AML patients, whereas Fr. III were increased, and there were no significant changes in Fr. I. (Fig. 1D-J). Therefore, defining suppressive or nonsuppressive FoxP3^+^ subpopulations by CD45RA might provide a true functional status of Treg cells and help to investigate the heterogeneity of Treg cells in leukemia.

### Fr. II cells in the peripheral blood of AML patients exhibit enhanced immunosuppressive function

**Fig. 2:**
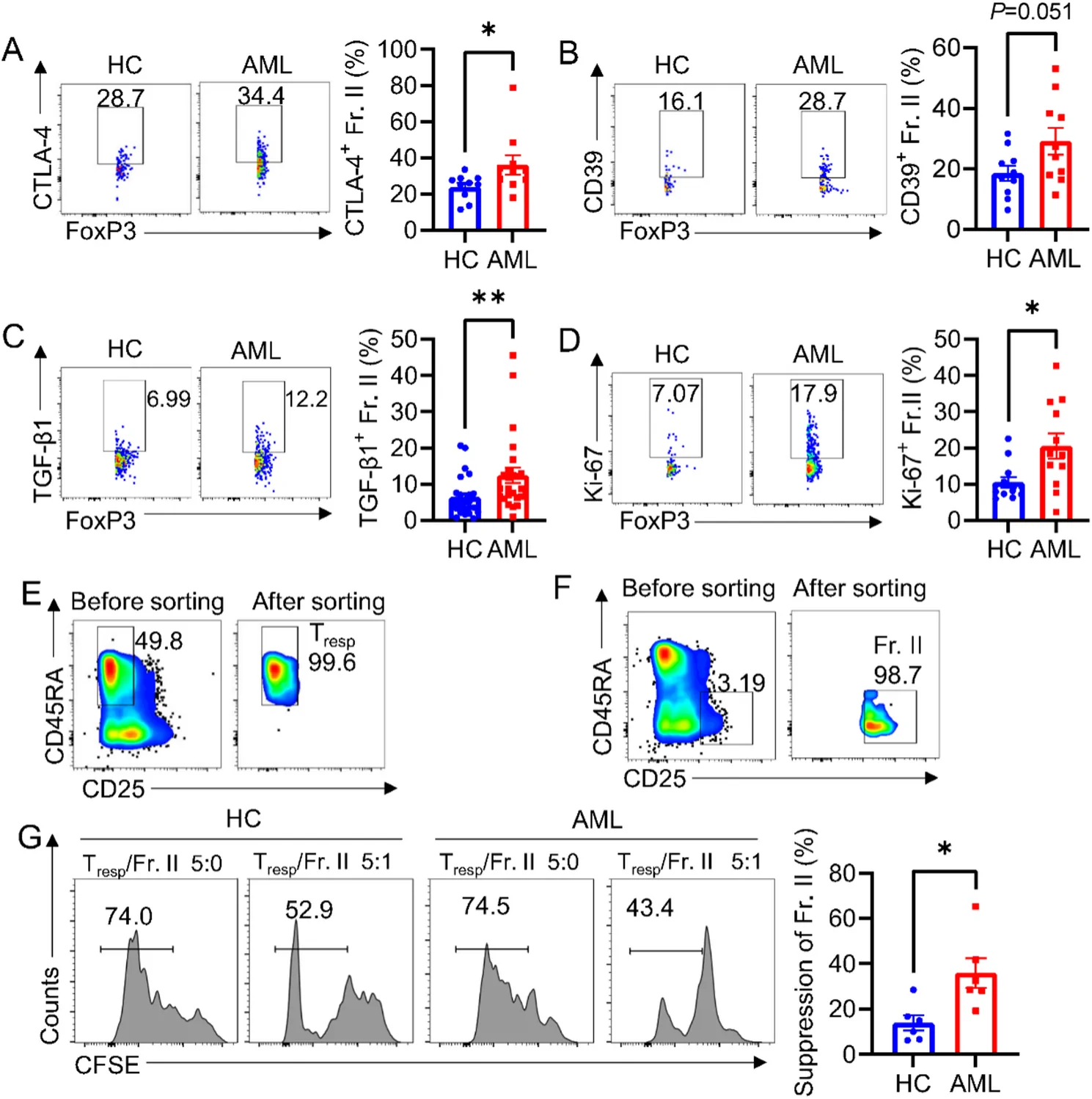
Fr. II cells from AML patients are more suppressive than those from healthy individuals. **A**-**D **Representative flow cytometry plots and percentages of (**A**) CTLA-4^+^ Fr. II, (**B**) CD39^+^ Fr. II, (**C**) TGF-β1^+^ Fr. II, and (**D**) Ki-67^+^ Fr. II in HCs and AML patients (A-B, *n* = 10 in each group; C, *n* = 25 in each group; D, *n* = 12 in each group). **E**, **F **Representative flow cytometry graphs of sorting efficiency for (**E**) CD25^−^CD45RA^+^CD4^+^ T cells (Tresp) and (**F**) CD25^+++^CD45RA^−^CD4^+^ T cells (Fr. II). **G **Evaluation of CFSE dilution in labeled Tresp following coculture with/without Fr. II cells at a 5:1 ratio for 5 days of anti-CD3/28 stimulation. Representative flow cytometry graphs and the percentage of Tresp cell proliferation are shown on the left. The statistical histogram depicting Fr. II suppression in HCs and AML patients is shown on the right (*n* = 6 in each group). The suppressive capacity of Fr. II toward T cells was calculated via the following formula: [100 × (1 - %CFSE^low^CD4^+^CD25^−^ T cells in coculture of %CFSE^low^CD4^+^CD25^−^ T cells alone)]. Tresp: responder T cells. The data are expressed as the means ± SEM. Either the Student’s *t* test or the Mann–Whitney *U* test was performed according to the data distribution. *P < 0.05; **P < 0.01

Furthermore, we detected cytokines expressed by Fr. I and Fr. II, both of which are suppressive. Compared with the HC group, the expression of CTLA‑4, CD39, and TGF‑β by Fr. II was higher in AML patients (Fig. 2A–C). Despite the reduced frequency and elevated late apoptosis ratio of Fr. II in AML patients (Figure S1A), these cells exhibited enhanced proliferative capacity, as reflected by increased Ki-67 expression (Fig. 2D). HC and AML group had comparable levels of early apoptosis (Figure S1B). In addition, expression of the chemokine receptor CXCR4, which mediates Treg migration to the bone marrow, was increased in AML (Figure S1C). These findings suggest that the reduced frequency of Fr. II may be related to both apoptosis and migration. No differences were detected in the levels of CTLA‑4, CD39, TGF‑β, and Ki-67 expressed by Fr. I between the AML patient group and the HC group (Figure S1D-G). Fr. III expressed lower levels of IFN-γ in the AML patient group, and comparable levels of IL-2 and Ki-67 compared with the HC group (Figure S1H-J).

The suppressive activity of Fr. II was assessed by its ability to inhibit the proliferation of autologous responder T cells. Previous studies have shown that the expression level of FoxP3 is positively proportional to that of CD25 in FoxP3^+^ subpopulations and sorted Fr. II as CD25⁺⁺⁺CD45RA⁻CD4⁺ [9, 12]. Therefore, we sorted responder T cells (Tresp, CD25⁻CD45RA^+^CD4^+^) and Fr. II, cocultured them at a ratio of 5:1 and monitored Tresp proliferation via CFSE labeling (Fig. 2E-F). The suppressive potency of Fr. II cells from AML patients was significantly greater than that of those from HCs (Fig. 2G). Taken together, the above results demonstrate that Fr. II in the peripheral blood of AML patients has increased immunosuppressive effects.

### Fr. II cells, with a Th17-like phenotype, are significantly increased in AML patients

**Fig. 3:**
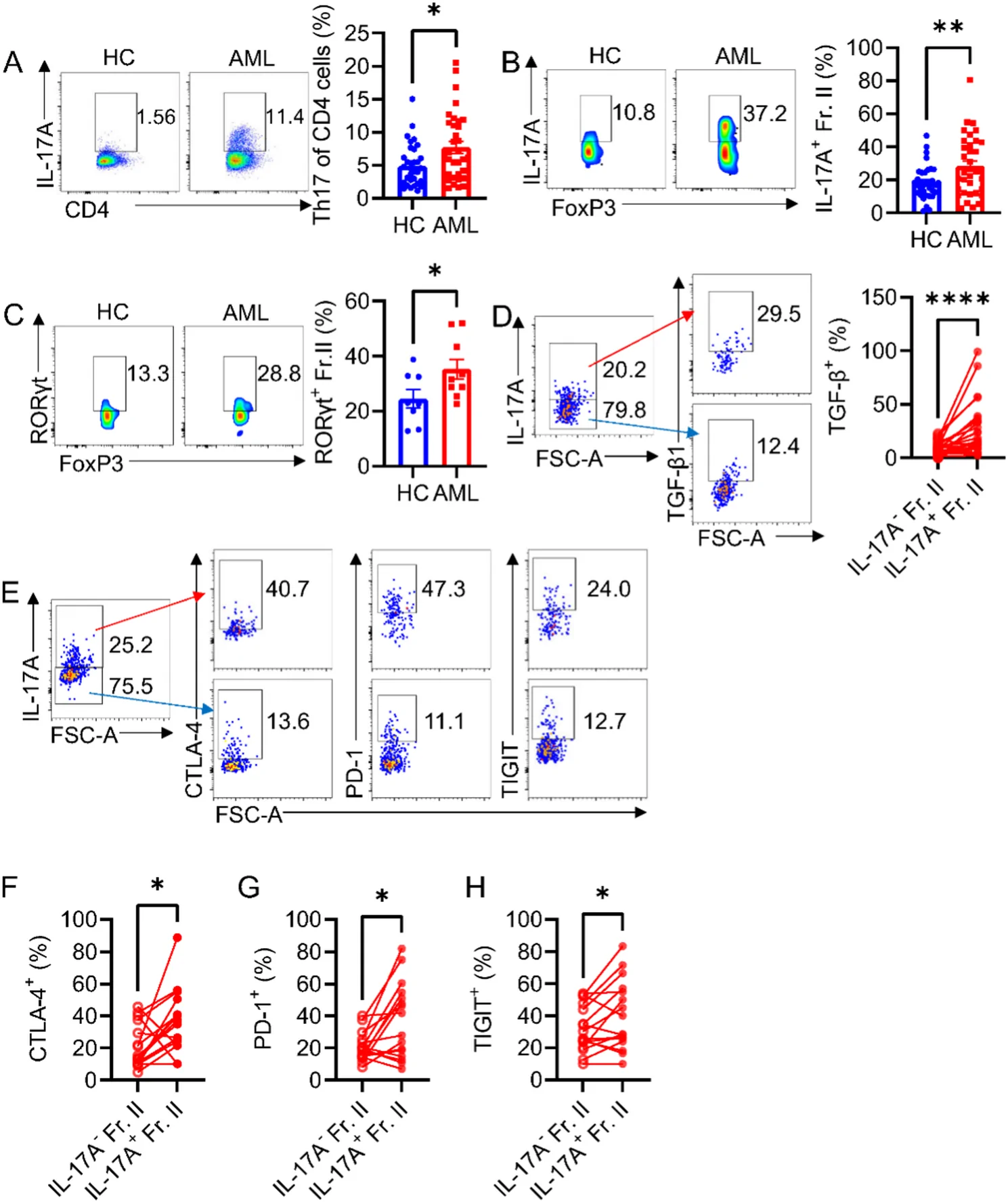
IL-17 A^+^ Fr. II cells are significantly increased in AML patients with elevated suppressive activity. **A **Representative flow cytometry plots and the percentages of CD4^+^IL-17 A^+^ T cells in HCs and AML patients (*n* = 34 in each group). **B**, **C **Representative flow cytometry plots and percentages of (**B**) IL-17 A^+^ Fr. II and (**C**) RORγt^+^ Fr. II in HCs and AML patients (**B**, *n* = 34 in each group; **C**, *n* = 8–9 in each group). The data are expressed as the means ± SEM. Either the Student’s *t* test or the Mann–Whitney *U* test was performed according to the data distribution. **D**-**H **Representative flow cytometry plots and the expression levels of (**D**) TGF-β1, (**E**, **F**) CTLA-4, (**E**, **G**) PD-1, and (**E**, **H**) TIGIT in IL-17 A^+^ and IL-17 A^−^ Fr. II cells of AML patients (**D**, *n* = 27 in each group, F-H, *n* = 16 in each group) are presented. Red arrows: cells gated from IL-17 A^+^ Fr. II cells; blue arrows: cells gated from IL-17 A^−^ Fr. II cells. Each line represents an individual sample. Statistical analyses were performed via paired *t* tests. *P < 0.05; **P < 0.01; ****P < 0.0001

Tregs have been identified as exhibiting plasticity. Tregs can convert into IL-17-producing Tregs. IL-17-producing Tregs can subsequently be further converted into Th17 cells [16, 22, 23]. To investigate whether the decreased proportion and absolute count of Fr. II cells are associated with imbalanced differentiation toward Th17-like cells, we analyzed IL-17 A^+^ levels in three FoxP3^+^CD4^+^ subsets. First, we found that the proportion of total Th17 cells in AML patients was greater than that in HCs (Fig. 3A). Moreover, the level of IL-17 A expressed by Fr. II was increased, and the Th17 differentiation-related transcription factor RORγt also tended to increase (Fig. 3B-C). IL-17 A and RORγt expression by Fr. I did not differ between the AML and HC groups (Figure S2A-B). The percentage of IL-17 A^+^ Fr. III cells did not change, whereas the level of RORγt expressed by Fr. III was elevated in AML patients (Figure S2C-D). These findings indicate that Fr. II cells exhibit propensity to express Th17-related cytokines.

We further characterized the suppressive function of IL-17 A^+^ Fr. II cells in AML patients. Compared with IL-17 A^−^ fraction II subsets, IL-17 A^+^ Fr. II cells displayed significantly higher expression of the inhibitory molecules TGF-β, CTLA-4, PD-1 and TIGIT (Fig. 3D-H). These findings indicate that IL-17 A^+^ Fr. II cells exert enhanced immunosuppressive effects.

### *In vitro* IL-6 neutralization hinders the Th17-like expression potential of Fr. II in AML

**Fig. 4:**
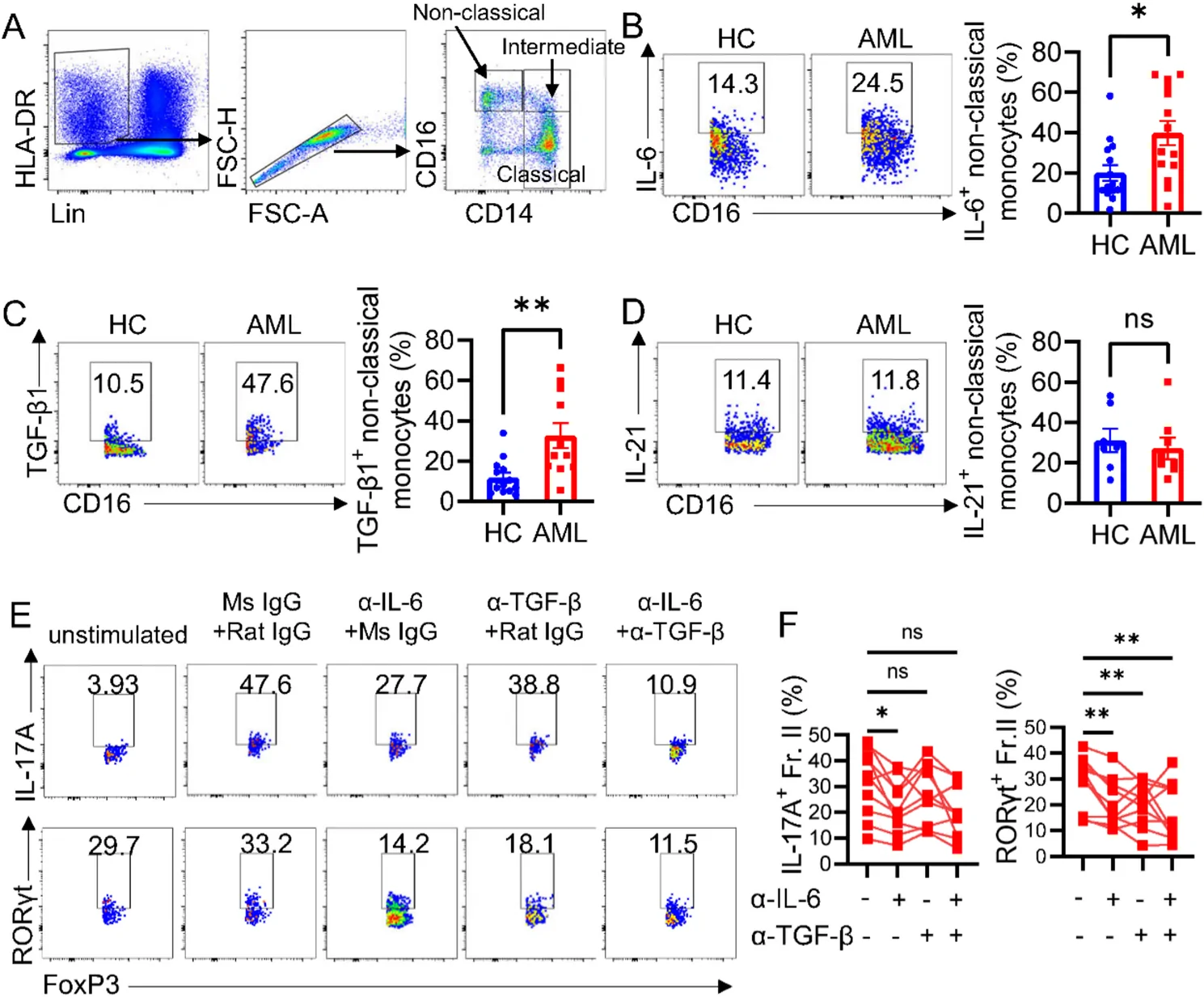
In vitro IL-6 neutralization inhibits the shift of Fr. II into Th17-like cells in AML. **A **The gating strategies for three subsets of monocytes: Lin (CD3, CD19, CD20, CD56, and CD66b)⁻HLA-DR⁺ cells were categorized into classical monocytes (CD14⁺CD16⁻), intermediate monocytes (CD14⁺CD16⁺) and non-classical monocytes (CD14⁻CD16⁺). **B**-**D **Representative flow cytometry plots and statistical histograms of (**B**) IL-6, (**C**) TGF-β1, and (**D**) IL-21 secreted by non-classical monocytes from HCs and AML patients (**B**, *n* = 14–15 in each group; **C**, *n* = 11–13 in each group; **D**, *n* = 7–8 in each group). The data are expressed as the means ± SEM. Either the Student’s *t* test or the Mann–Whitney *U* test was performed according to the data distribution. **E**, **F **PBMCs from AML patients were cultured for 72 h under five conditions: unstimulated (without anti-CD3/28); or stimulated with anti-CD3/28 in the presence of isotype control, 10 µg/mL anti-IL-6, 10 µg/mL anti-TGF-β, or their combination. **E **Representative flow cytometry plots of Fr. II cells expressing IL-17 A and RORγt after corresponding antibody neutralization in AML patients are shown. **F **Statistical graph showing IL-17 A^+^ Fr. II and RORγt^+^ Fr. II cells after the corresponding neutralizing antibodies were added to the PBMCs of AML patients (*n* = 10). Each line corresponds to one individual. One-way *ANOVA* was used. *P < 0.05; **P < 0.01; ns, not significant

Studies have shown that monocytes are involved in the generation of IL-17-producing Tregs and the maintenance of the Th17/Treg balance [18, 24, 25]; therefore, we further explored the reciprocal regulation between monocytes and Fr. II in AML patients. Monocytes were divided into three subsets: classical monocytes (CD14⁺CD16⁻), intermediate monocytes (CD14⁺CD16⁺), and non-classical monocytes (CD14⁻CD16⁺) (Fig. 4A). IL-6, IL-21 and low doses of TGF-β can promote the expression of RORγt, which can promote Th17 development [26, 27]. We further investigated the expression of Th17 differentiation-associated cytokines in monocyte subsets. The results revealed that the levels of IL-6 and TGF-β expressed by non-classical monocytes increased significantly, whereas the level of IL-21 did not change (Fig. 4B-D). No differences were observed in classical or intermediate monocytes (Figure S3). The above results indicate that non-classical monocytes have the potential to promote the differentiation of Fr. II into Th17-like cells in AML patients.

Next, to understand the role of IL-6 and TGF-β in the acquisition of Th17-like phenotype of activated Tregs, we stimulated PBMCs isolated from AML patients with anti-CD3/28 in the presence of neutralizing antibodies against IL-6 and/or TGF-β. The results revealed that upon IL-6 neutralization, the Th17-like shift of Fr. II in AML patients was reduced, as evidenced by decreased expression of IL-17 A and RORγt (Fig. 4E-F). The experiments also suggested that in vitro TGF-β neutralization reduced the percentage of RORγt^+^ Fr. II but that the percentage of IL-17 A^+^ Fr. II did not change. Our results indicate that IL-6 plays a key role in mediating the acquisition of a Th17‑like phenotype by Fr. II in AML patients.

### The IL-6/JAK2/STAT3 axis drives the shift from Fr. II to Th17-like development

**Fig. 5:**
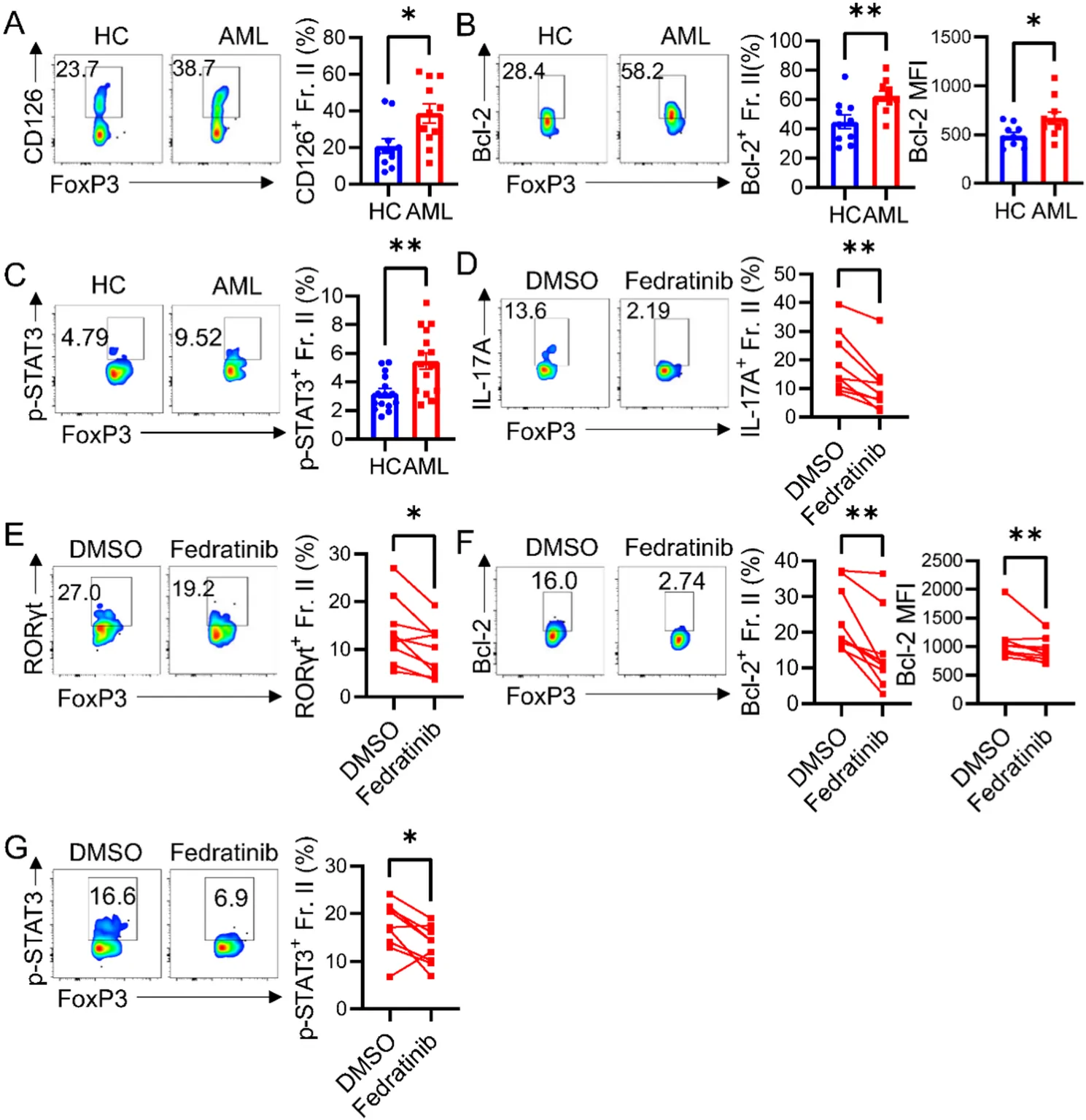
JAK2 inhibitor impairs the generation of IL-17 A^+^ Fr. II in AML patients. **A**-**C **Representative flow cytometry plots and quantitative analyses in HCs and AML patients: (**A**) percentages of CD126^+^ Fr. II, (**B**) frequencies of Bcl-2^+^ Fr. II and mean fluorescence intensity (MFI) of Bcl-2 in Fr. II, (**C**) percentages of p-STAT3^+^ Fr. II. (**A**, *n* = 11 in each group; **B**, *n* = 10 in each group; **C**, *n* = 15 in each group). The data are expressed as the means ± SEM. Either the Student’s *t* test or the Mann–Whitney *U* test was performed according to the data distribution. **D**-**G **PBMCs isolated from AML patients were stimulated with anti-CD3/28 in vitro and treated with 100 nM Fedratinib (JAK2 inhibitor) or DMSO for 72 h. Representative flow cytometry plots and quantifications in HCs and AML patients: percentages of (**D**) IL-17 A^+^ Fr. II, (**E**) RORγt^+^ Fr. II, and (**G**) p-STAT3^+^ Fr. II, (**F**) frequencies of Bcl-2^+^ Fr. II and MFI of Bcl-2 in Fr. II (*n* = 9 in each group). Each line represents an individual sample. Statistical analyses were performed via paired *t* tests. *P < 0.05; **P < 0.01

To further investigate how IL-6 mediates the development of Fr. II toward a Th17-like phenotype, we investigated the IL-6/JAK2/STAT3 pathway, which plays a critical role in the Th17/Treg balance [28–30]. Compared with those in the HCs, Fr. II in the peripheral blood of AML patients exhibited elevated levels of IL-6 receptor (CD126), increased phosphorylated of STAT3 and enhanced the expression of Bcl-2, which is an activation marker of the IL-6/JAK2/STAT3 pathway (Fig. 5A-C).

To verify the role of the JAK2 pathway in the shift of Fr. II into a Th17-like phenotype, in vitro blockade experiments were conducted by treating isolated PBMCs from AML patients with a specific JAK2 inhibitor. The results revealed that the JAK2 inhibitor significantly suppressed the Th17-like differentiation potential of Fr. II cells, as evidenced by decreased expression of IL-17 A, RORγt, Bcl-2 and p-STAT3 in Fr. II cells (Fig. 5D-G). These findings reveal that Fr. II cells differentiate into Th17-like cells in AML patients via the IL-6/JAK2/STAT3 pathway.

### IL-6 secreted by non-classical monocytes of AML patients induces Fr. II differentiation into a Th17-like phenotype via activation of the JAK2/STAT3 pathway

**Fig. 6:**
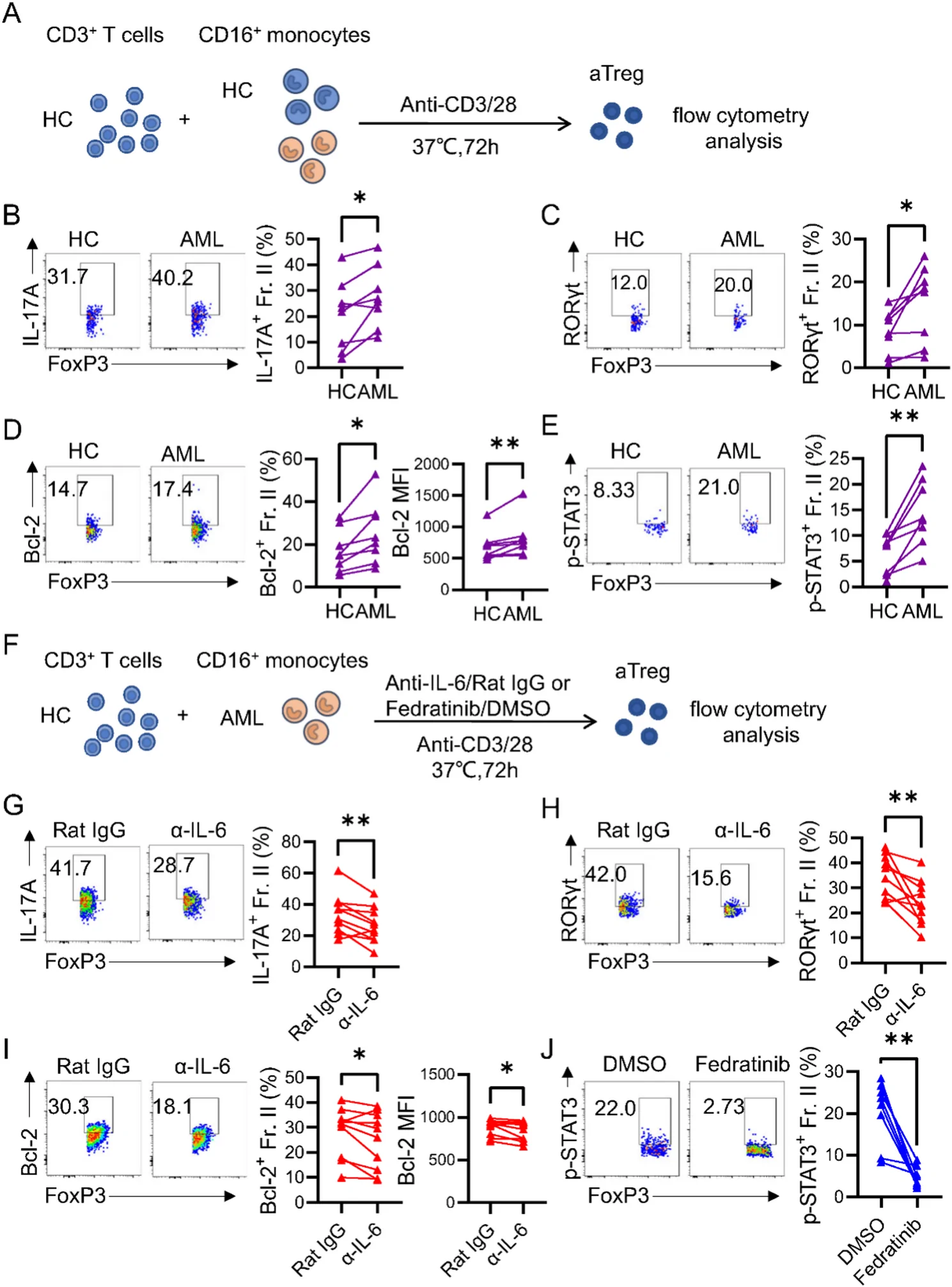
Non-classical monocytes drive the development of Fr. II into a Th17-like phenotype via the IL6/JAK2/STAT3 axis in AML. **A **Schematic representation of the experimental process. CD3⁺ T cells isolated from HCs were cocultured with non-classical monocytes sorted from HCs or AML patients at a 10:1 ratio and stimulated with anti-CD3/CD28 antibodies for 3 days. Flow cytometry was used to detect Fr. II in CD3^+^ T cells. **B**-**E **Representative flow cytometry plots and statistical analyses showing the frequencies of Fr. II cells expressing (**B**) IL-17 A, (**C**) RORγt, and (**E**) p-STAT3, as well as (**D**) the frequency of Bcl-2^+^ Fr. II and MFI of Bcl‑2 in Fr. II cells (*n* = 8 in each group). **F **Schematic overview of the experimental workflow. HC-derived CD3⁺ T cells were cocultured with non-classical monocytes sorted from AML patients at a 10:1 ratio with anti-CD3/28 stimulation. **G**-**J **Cocultured cells were treated with 10 µg/mL IL-6 neutralizing antibody or Rat IgG for 72 h. Representative flow plots and statistical analyses showing the frequencies of Fr. II cells expressing (**G**) IL-17 A, (**H**) RORγt, as well as (**I**) the frequency of Bcl-2^+^ Fr. II and MFI of Bcl-2 in Fr. II cells (*n* = 10). **J **Cocultured cells were treated with 100 nM Fedratinib or DMSO for 72 h. Representative flow plots and statistical analyses of p-STAT3⁺ Fr. II are shown (*n* = 8). Each line represents an individual sample. Statistical analyses were performed via paired *t* tests. *P < 0.05; **P < 0.01

To evaluate whether elevated IL-6 expression in non-classical monocytes promotes Fr. II differentiation into a Th17-like phenotype, we first cocultured non-classical monocytes freshly sorted from HCs or AML patients with allogeneic CD3⁺ T cells sorted from HCs (Fig. 6A). Compared with HC-derived monocytes, non-classical monocytes isolated from AML patients exhibited a greater capacity to promote the occurrence of Th17-like Fr. II via the IL-6/JAK2/STAT3 axis. This was evidenced by increased expression of IL-17 A, RORγt, Bcl-2 and p-STAT3 in Fr. II cells cocultured with non-classical monocytes from AML patients (Fig. 6B-E).

We subsequently performed in vitro IL-6 neutralization in coculture systems of HC-derived CD3⁺ T cells and AML-derived non-classical monocytes (Fig. 6F). IL-6 neutralization inhibited the activation of the JAK2/STAT3 pathway and the development of Fr. II toward a Th17-like phenotype, as demonstrated by decreased expression of IL-17 A, RORγt and Bcl-2 in Fr. II (Fig. 6G-I). The above data demonstrate that IL-6 expressed by non-classical monocytes promotes the generation of IL-17 A^+^ Fr. II. Consistently, the JAK2 inhibitor suppressed p-STAT3 expression of Fr. II cells in CD3⁺ T cells cocultured with AML-derived non-classical monocytes (Fig. 6F, J). These results suggest that non-classical monocytes from AML patients can induce Th17-like shift of Fr. II by activating the IL-6/JAK2/STAT3 pathway.

### The shift from Fr. II to Th17-like development aggravates disease severity

**Fig. 7:**
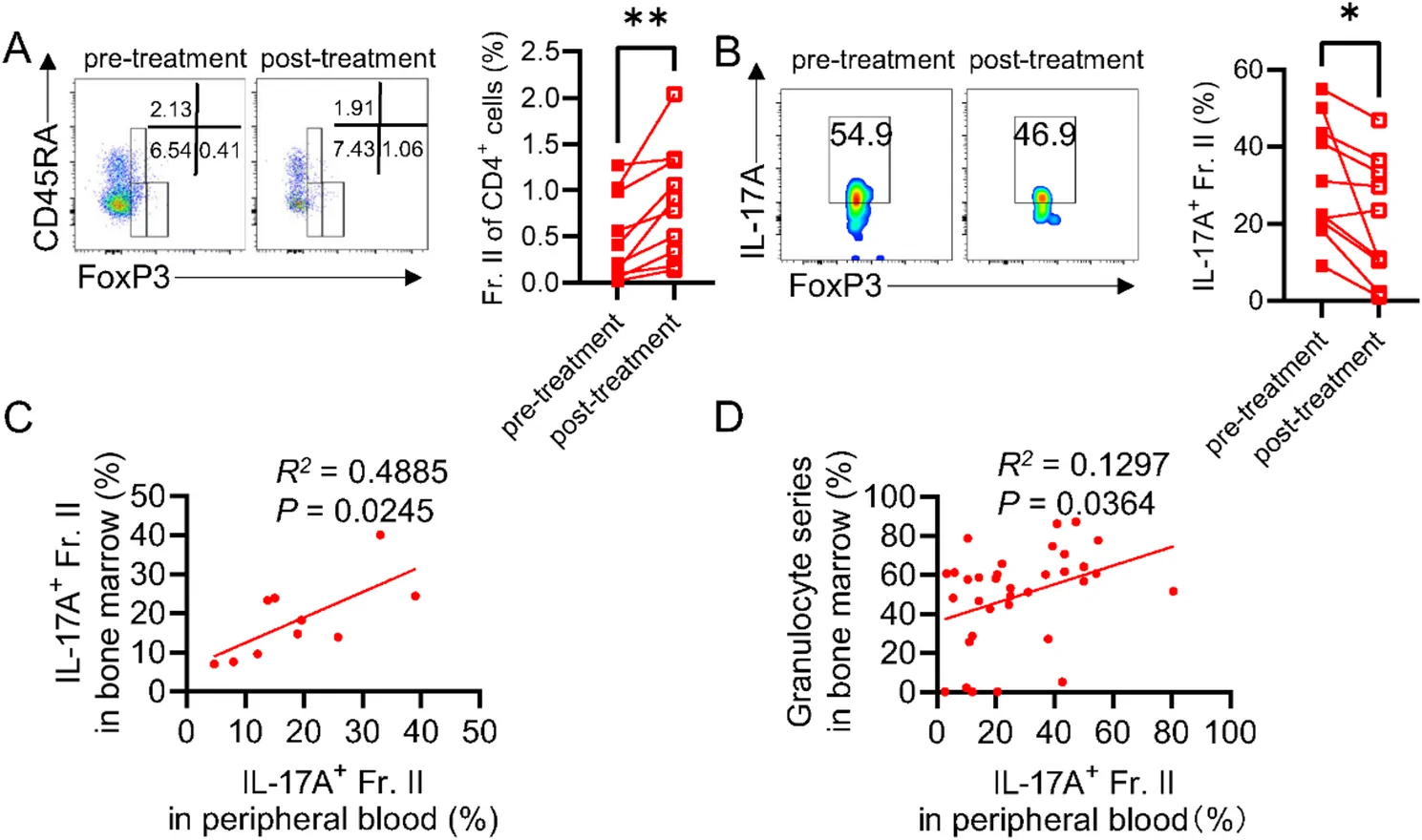
IL 17 A^+^ Fr. II are associated with the progression of acute myeloid leukemia. **A**, **B **Representative flow cytometry plots and statistical analyses depicting changes in the percentages of Fr. II and IL-17 A^+^ Fr. II in PBMCs from AML patients at pre-treatment and post-treatment recovery stages (*n* = 10). Each line represents an individual sample. Statistical analyses were performed via paired *t* tests. *P < 0.05; **P <0.01. **C **Correlation between the percentage of IL-17 A⁺ Fr. II in the peripheral blood and bone marrow (*n* = 10). **D **Correlation between the percentage of IL-17 A⁺ Fr. II in the peripheral blood and the proportion of granulocyte series in the bone marrow (*n* = 34). Coefficient of determination (*R*^*2*^), corresponding p values (*P*), and linear trendlines are depicted in each plot

To assess the phenotypic changes of Fr. II associated with clinical recovery, we analyzed pre- and post-treatment peripheral blood from AML patients (clinical information is summarized in Supplementary Table 2). We observed an increase in the percentage of Fr. II in patients during post-treatment recovery (partial remission in the bone marrow), accompanied by a concurrent decrease in the percentage of IL-17 A⁺ Fr. II (Fig. 7A-B). These findings suggest that the IL-17 A⁺ Fr. II may correlate with disease activity.

In addition, we detected IL-17 A expression in Fr. II isolated from the bone marrow of AML patients. Given that bone marrow specimens from HCs were unavailable, we analyzed the correlation between the expression of IL-17 A by Fr. II cells in the peripheral blood and bone marrow of AML patients and confirmed a significant positive correlation (Fig. 7C). Furthermore, IL-17 A^+^ Fr. II in peripheral blood was also positively correlated with the proportion of bone marrow granulocyte series, suggesting that its elevation is associated with AML-related myelogram abnormalities and disease progression (Fig. 7D). In conclusion, high expression of IL-17 A on Fr. II may be a marker of poor prognosis in AML patients.

## Discussion

Our research reveals that Fr. II, which play a major suppressive role, are significantly reduced in the peripheral blood of AML patients. However, their inhibitory function is enhanced. In particular, IL-6 secreted by non-classical monocytes induces Fr. II to develop a Th17-like phenotype by activating the JAK2/STAT3 pathway, and this phenotypic shift is correlated with the severity of AML. Therefore, Th17-like Fr. II may be used as a research target for immunotherapy.

Consistent with the results of previous studies, we found that the proportion of CD25^+^FoxP3^+^CD4^+^ T cells in the peripheral blood of AML patients was increased [6]. However, human FoxP3^+^CD4^+^ T cells include both inhibitory (Fr. I and Fr. II) and noninhibitory (Fr. III) subsets. We found that in the peripheral blood of AML patients, the proportion of Fr. II decreased, the level of Fr. I remained unchanged, and Fr. III increased. However, Fr. II accounted for the smallest proportion among the three groups, while Fr. III accounted for the largest proportion. There are three possible reasons for the decrease in Fr. II. Firstly, the activation of STAT3, a key factor in Th17 development, suppressed the expression of Foxp3, a major regulator of Fr. II development in T cells [31]. Some Fr. II transform into IL-17 A^+^ Fr. II intermediates and then differentiate into Th17 cells [22]. Secondly, Fr. II in peripheral blood can migrate to the bone marrow through CXCL12/CXCR4 signaling [32].Thirdly, late apoptosis of Fr. II was significantly increased. Although Fr. II number is decreased, their increased suppressive ability implies that Fr. II may be better indicators of suppressive function than FoxP3^+^CD4^+^ Tregs in a variety of diseases.

AML patients have insufficient production of red blood cells and platelets in the peripheral blood due to abnormal bone marrow hematopoietic function [33]. For clinical safety reasons, the western blotting method, which consumes a relatively large amount of peripheral blood, is not recommended for verifying the JAK2/STAT3 pathway to avoid increasing the risk of anemia and bleeding. Therefore, we used flow cytometry (FACS) as an alternative method for relevant detection, as FACS has lower sample requirements. The present study focuses only on the JAK2/STAT3 signaling pathway. Given that activation of the IL-6 pathway cross-regulates downstream transcriptional programs with other STAT family members [34], assessing the phosphorylation levels of other STAT molecules, including STAT1, STAT2, STAT5, and STAT6, is also of great significance. Bcl-2, serves as a marker reflecting the activation status of the JAK2/STAT3 signaling pathway [35]. Of note, venetoclax, which is widely used in clinical settings, functions as a selective Bcl-2 inhibitor. In Fig. 7A-B, more than half of the patients were treated with venetoclax in combination with other drugs. The treatment with venetoclax has been shown to alleviate disease progression, indirectly supporting the notion that aberrant activation of JAK2/STAT3 contributes to AML severity, which is consistent with our findings.

The role of Th17 cells in tumor progression remains controversial [36, 37]. Th17 cells are increased in patients with chronic lymphocytic leukemia and are associated with a longer survival period [38]. During the resolution stage of *Mycoplasma pneumoniae* (*MP*) infection, the impaired Treg function was associated with an increase in IL-17 A^+^ Tregs and Th17 cells, and remained impaired even 1 year after *MP* infection [18]. However, AML patients with a high proportion of Th17 cells have a poor prognosis, whereas those with a high proportion of Th1 cells have a longer survival period [39]. IL-17^+^FoxP3^+^ T cells in rheumatoid arthritis (RA) are involved in the pathogenesis of RA, highlighting the important pathological significance of FoxP3 instability in the generation of pathogenic Th17 cells [40]. Furthermore, Th17 cells can be transformed into IL-17/IL-10-secreting cells, creating an environment with reduced immune control, which may promote infection and potentially lead to leukemia immune escape [19]. These findings further indicate that Th17 cells play a key role in the pathogenesis of AML. Compared with pre-treatment patients, we observed a marked reduction in the percentage of IL‑17 A⁺ Fr. II cells in AML patients during post-treatment recovery. These findings suggest that elevated Th17‑cell levels may correlate with disease severity in AML. The immune balance between Th17 cells and Fr. II may become an important therapeutic target and prognostic predictor for AML patients.

Previous studies have shown that IL-6 secreted by mesenchymal stromal cells (MSCs) in AML patients plays an important role in the process of AML. On one hand, MSCs provide pro-survival effects for leukemia blasts and supports the long-term maintenance of leukemia stem cells (LSCs) [41]. On the other hand, secreted bone morphogenetic protein 6 (BMP6) in MSCs can promote the secretion of IL-6 and further facilitate the differentiation of leukemic cells [42]. Moreover, in vitro studies have shown that IL-6 secreted by MSCs activates the JAK2/STAT3 pathway in AML cells and induces epithelial-mesenchymal transition (EMT) -like procedures, which provides a therapeutic target for AML chemotherapy [43]. Lu Dejin et al. demonstrated that MSCs could transform monocytes into anti-inflammatory phenotypes [44], and induce monocytes to enter the bloodstream [45]. Our research only showed that IL-6 secreted by non-classical monocytes in peripheral blood promotes the differentiation of Fr. II cells into Th17 cells. The influence of MSCs-derived IL-6 in bone marrow still needs further research to be clarified.

Our results indicate that the proportion of RORγt^+^ Fr. II among stimulated PBMCs from AML patients also decreased after TGF-β neutralization in vitro. The role of TGF-β in Th17 development remains controversial. In murine experimental settings, evidence indicates that TGF-β1 is responsible for the differentiation of naive T cells into Th17 cells [46], but Th17 cell differentiation can also occur when TGF-β signaling is lacking and when IL-6 or IL-23 is applied in combination with IL-1β [47]. High levels of TGF-β1 have been detected in the bone marrow microenvironment of AML patients [27]. Studies that used serum-free cultured neonatal CD4⁺ T cells in humans revealed that exogenous TGF-β supplementation is necessary for Th17 cell differentiation [48, 49]. However, in AML patients, analyses of in vivo PBMCs and bone marrow mononuclear cells (BMMCs) revealed that IL-1β, IL-6, and IL-23 (rather than TGF-β1) play crucial roles in Th17 cell induction [39]. Therefore, further research is still needed on the role of TGF-β1 in Th17 cells in the tumor microenvironment of AML patients.

The differentiation of CD4^+^ T towards Th17 involves the joint participation of multiple cytokines. IL-6 can activate STAT3, thereby inducing RORγt expression and stimulating T cells to produce IL-17 A [50, 51]. Notably, several studies have proposed that naive T cells differentiate into Th17 and Treg cells in an interlaced pattern, as TGF-β favors Treg cell development, whereas TGF-β and IL-6 facilitate Th17 cell differentiation, which accounts for the plasticity of these two cell subsets [15, 26, 52]. In the disease model of ulcerative colitis, the increase of IL-6 leads to the excessive activation of Th17 and the impaired function and reduced counts of Treg [28]. IL-6 drives RA progression [53], while the conversion of FoxP3^+^ T cells to Th17 cells was also observed in the synovial tissue of patients with active RA [40]. In fact, in many diseases, the increase of IL-6 can lead to the transformation of Treg into Th17. In our study, we cocultured non-classical monocytes sorted from HCs or AML patients with allogeneic CD3⁺ T cells sorted from HCs. Compared with HC-derived monocytes, AML-derived non-classical monocytes exhibited a greater capacity to promote the occurrence of Th17-like Fr. II. In vitro IL-6 neutralization in coculture systems of HC-derived CD3⁺ T cells and AML-derived non-classical monocytes leads to a decrease in the proportion of IL-17 A^+^ Fr. II. Therefore, we speculate that Fr. II in healthy individuals may also undergo similar transformation upon ex vivo IL‑6 stimulation. The interactions among other cytokines still require further study in the future.

In conclusion, we revealed that among the three functionally and phenotypically heterogeneous FoxP3⁺CD4⁺ subsets in the peripheral blood of AML patients, although the proportion of Fr. II is decreased, their suppressive function is enhanced. Additionally, the occurrence of a cascade reaction to induce the conversion of Fr. II to a Th17-like phenotype by non-classical monocytes is correlated with poor prognosis. Our research identified a potential valuable immune target for guiding the treatment of AML patients.

## Supplementary Information

Supplementary Material 1.

## Data Availability

All data supporting the research conclusions are available upon request from the corresponding author.

## Abbreviations

AML: Acute myeloid leukemia
HC: Healthy control
Tregs: Regulatory T cells
Tresp: Responder T cells
LSCs: Leukemia stem cells
MDSCs: Myeloid-derived suppressor cells
BMMCs: Bone marrow mononuclear cells
