# Supplementary material for: IL-6/JAK2/STAT3 axis mediates non-classical monocyte-induced Th17-like conversion of activated Tregs in acute myeloid leukemia patients
Source: BMC Cancer. 2026 Jul 28;26:1319. doi: 10.1186/s12885-026-16619-6 (PMC13721268; doi:10.1186/s12885-026-16619-6)
Supplement: Supplementary file 1 [file 12885_2026_16619_MOESM1_ESM.docx]

**Supplementary table**

**Supplementary Table 1: the clinical parameters of total HC and AML patients enrolled in this study.**

| **Clinical characteristics** | **AML** | **HC** | ***P*** |
| --- | --- | --- | --- |
| N | 73 | 62 |  |
| Sex (male/female) | 31/42 | 28/34 |  |
| Age (years) | 55.00 (48.00-62.5) | 51.50 (45.00-58.25) | ns |
| WBC (×10^9^/L) | 4.46 (2.50-6.37) | 5.74 (4.87-6.82) | <0.01 |
| NEUT# (×10^9^/L) | 2.22 (0.77-3.65) | 3.30 (2.76-3.89) | <0.001 |
| LYMPH# (×10^9^/L) | 1.10 (0.79-1.60) | 1.84 (1.59-2.35) | <0.0001 |
| MONO# (×10^9^/L) | 0.46 (0.29-0.67) | 0.34 (0.30-0.40) | <0.001 |
| EO# (×10^9^/L) | 0.03 (0.01-0.15) | 0.12 (0.07-0.20) | <0.0001 |
| BASO# (×10^9^/L) | 0.01 (0.01-0.03) | 0.03 (0.02-0.05) | <0.0001 |
| HGB (g/L) | 106.00 (92.50-125.50) | 139.50 (130.80-154.50) | <0.0001 |
| PLT (×10^9^/L) | 121.00 (57.00-200.00) | 214.50 (188.00-241.00) | <0.0001 |

**Note:** Data are presented as median (interquartile range, 25 to 75). Significance was assessed by the Mann–Whitney *U* test. AML, acute myelogenous leukemia; HC, healthy control; WBC: white blood cells; NEUT#, neutrophil count; LYMPH#, lymphocyte count; MONO#, monocyte count; EO#, eosinophil count; BASO#, basophil count; HGB, hemoglobin; PLT, platelets.

**Supplementary Table 2: pre‑ and post‑treatment information of AML patients in Figure 7.**

| **AML patients** | **Gender** | **Age** | **Chemotherapeutic and targeted agents** |
| --- | --- | --- | --- |
| 1 | M | 80 | AZA + VEN |
| 2 | F | 57 | Ara-C + VEN + DNR |
| 3 | M | 45 | Ara-C + VEN + DNR + DEC |
| 4 | F | 32 | Ara-C + VEN |
| 5 | F | 55 | ATRA + ATO |
| 6 | M | 24 | Ara-C + VEN |
| 7 | F | 48 | Ara-C + DNR |
| 8 | F | 18 | IDA + Ara-C |
| 9 | M | 56 | IDA + Ara-C |
| 10 | M | 74 | AZA + VEN |

**Note:** AZA: azacitidine; VEN: venetoclax; Ara-C: cytarabine; DNR: daunorubicin; DEC: decitabine; ATRA: all-trans retinoic acid; ATO: arsenic trioxide; IDA: idarubicin.

**Supplementary Figures**

**Figure S1. The phenotypes of Fr. II and cytokines secreted by Fr. I and Fr. III in HC and AML patients.**

(A-H) The averaged percentages were shown for: (A) Annexin V^+^7-AAD^+^ Fr. II, (B) Annexin V^+^7-AAD^-^ Fr. II, (C) CXCR4^+^ Fr. II, (D) CTLA-4^+^ Fr. I, (E) CD39^+^ Fr. I, (F) TGF-β⁺ Fr. I, (G) Ki-67^+^ Fr. I, and (H) Ki-67^+^ Fr. III in HC and AML patients (A-B, n=9 in each group; C-E, n=12 in each group; F, n=27-31 in each group; G-H, n=12 in each group). (I-J) Representative flow cytometry plots and averaged percentages of Fr. III expressing (I) IFN-γ and (J) IL-2 in HC and AML patients (C, n=28-31 in each group; D, n=14 in each group). Data are expressed as the mean ± SEM. Either the Student’s *t*-test or Mann–Whitney *U* test was performed according to the data distribution. ***P*<0.01; ns, not significant.

**Figure S2. Th17-related cytokines and transcription factor expressed by Fr. I and Fr. III in HC and AML patients.**

Representative flow cytometry plots and averaged percentages of (A) IL-17A^+^ Fr. I, (B) RORγt^+^ Fr. I, (C) IL-17A^+^ Fr. III, and (D) RORγt^+^ Fr. III for HC and AML patients (A, n=21-23 in each group; B, n=8 in each group; C, n=23-24 in each group; D, n=8-9 in each group). Data are expressed as the mean ± SEM. Either the Student’s *t*-test or Mann–Whitney *U* test was performed according to the data distribution. **P*<0.05; ns, not significant.

**Figure S3. Neither classical nor intermediate monocytes from AML patients produce elevated levels of cytokines associated with Th17 cell differentiation.**

(A-C) The averaged percentages are shown for (A) IL-6, (B) TGF-β and (C) IL-21 secreted by classical monocytes in HC and AML patients (A, n=13-15 in each group; B, n=11-13 in each group; C, n=7-8 in each group). (D-F) The averaged percentages are shown for (D) IL-6, (E) TGF-β and (F) IL-21 secreted by intermediate monocytes in HC and AML patients (D, n=14-15 in each group; E, n=11-13 in each group; F, n=6-8 in each group). Data are expressed as the mean ± SEM. Either the Student’s *t*-test or Mann–Whitney *U* test was performed according to the data distribution. ns, not significant.
